# Genome-wide donor–recipient non-HLA mismatch and graft loss

**DOI:** 10.1093/ndt/gfaf073

**Published:** 2025-04-25

**Authors:** Josef Pickl, Andreas Heinzel, Stephen Shoebridge, Alexander Kainz, Rainer Oberbauer

**Affiliations:** Department of Nephrology, Medical University of Vienna, Vienna, Austria; Department of Nephrology, Medical University of Vienna, Vienna, Austria; Department of Nephrology, Medical University of Vienna, Vienna, Austria; Department of Nephrology, Medical University of Vienna, Vienna, Austria; Department of Nephrology, Medical University of Vienna, Vienna, Austria

**Keywords:** kidney, mismatch, non-HLA, transplantation

## Abstract

HLA-matching between donor (D) and recipient (R) is routinely performed in kidney allocation to optimize allograft survival but explains only a moderate variability of these outcomes. Recent findings suggest that donor-to-recipient mismatches outside the HLA region contribute to alloimmunity and graft loss, but the extent varies in different publications. We therefore conducted a systematic review of publications on this subject using a broad search string in our literature review in accordance with current guidelines for systematic reviews. The effect sizes were analyzed by a meta-analysis. A total of 1890 publications from 2019–25 within three different repositories (465 Medline, 1408 Embase, 17 Central) were systematically screened using the PICOTS (Population, Intervention, Comparator, Outcome, Time and Setting) system, which resulted in 12 eligible papers that met the inclusion criteria. Cohort studies that investigated the association of D-R non-HLA SNP mismatch and graft rejection/loss in renal transplant patients were included. We found that overall SNP mismatch between D-R pairs outside the HLA region was independently numerically associated with rejection hazard ratio (HR) 1.26 [95% confidence interval (CI) 0.97–1.65] and graft loss HR 1.35 (95% CI 0.86–2.12). Furthermore, loss of function mutation of the gene *LIMS1* in the recipient who received a transplant organ with at least one functioning copy (collision genotype) was numerically associated with rejection HR 1.23 (95% CI 0.68–2.23) and graft loss HR 1.43 (95% CI 0.61–3.36). The exact quantification of the effect size of these mismatches varied by publication and needs further investigation. Based on these data, the strength of immunosuppression may be guided by the load of D-R mismatches in the future.

## INTRODUCTION

The role of HLA compatibility in kidney transplantation has been well studied over the last decades and the practice of sufficiently matching donor–recipient (D-R) HLA-A, B and DR alleles has ameliorated overall kidney transplant survival significantly [1, 2]. Despite the considerable improvements of short-term allograft survival, sufficient long-term kidney transplant patency remains a challenge that has not yet been solved [3]. Although HLA matching has been proven to be the central pillar in organ allocation to facilitate reasonable allograft survival, non-HLA alloimmunity may also contribute to graft loss [4]. Modern approaches aim to improve D-R matching by considering HLA-eplet mismatch in the process of organ allocation. These more precise matching methods may allow for optimization of immunosuppression in the better-matched patients [5]. Recent studies went even further by suggesting that in HLA-matched D-R pairs, single nucleotide polymorphisms (SNPs), either in the donor or the recipient, could lead to rejection through various mechanisms which will be further discussed in the subsequent paragraphs. The objective of this quantitative review was to summarize the available evidence based on the association of D-R mismatch, both in general terms and at single genetic loci, with hard outcomes such as biopsy-confirmed graft rejection and graft loss in renal transplant patients.

## METHODS

**Figure 1:**
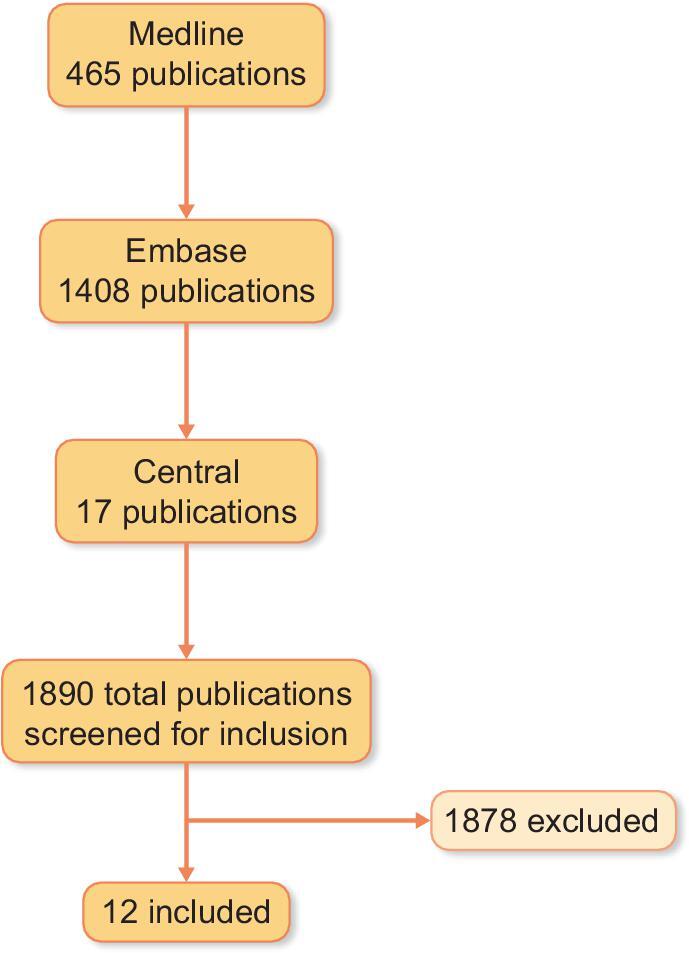
Screening and selection of publications. Included papers evaluated the associations between D-R non-HLA SNP mismatch and objective outcomes such as graft loss or biopsy-proven graft rejection.

**Figure 2:**
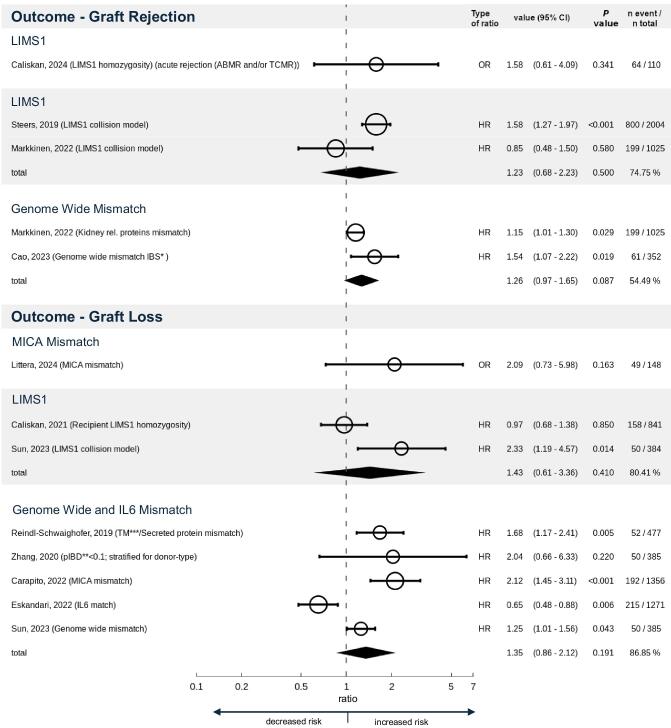
Meta-analysis. Study descriptions (left) with their respective HR/OR and CIs (middle) and *P*-values with total study participants and events (right). TM, transmembrane. The size of symbols (rings) corresponds to the number of events. Provided HRs are not comparable to provided Ors, and are therefore depicted in their respective rows in the figure.

First, the review question was defined according to the PICOTS (Population, Intervention, Comparator, Outcome, Time and Setting) system (Table 1) [6]. Subsequently, a structured literature search using a search string containing (13) kidney transplantation and (34) non-HLA related MeSH terms and key words was conducted with the help of two staff-librarians from the Medical University of Vienna. We constructed a broad search filter with the goal of maximum retrieval of studies as recommended by Ingui *et al*., which resulted in the identification of 1890 publications from 2019 to 2025 within three different repositories (465 Medline, 1408 Embase, 17 Central) [7]. After systematic screening in accordance with the PICOTS system to limit the risk of selection bias and following the recommendations by Riley *et al*. by screening the papers first by title, then by abstract, 12 eligible studies were selected for this review (Fig. 1) [6]. Of those 1878 papers not included, the majority did not investigate the D-R recipient genetic mismatch, but rather the association of either recipient or donor SNPs with graft outcomes and were therefore excluded. As previously stated, the reviewing process was of a systematic nature, however due to limitations (small sample size, heterogenous studies) and adhering to modern standards we were only able to conduct an extenuated meta-analysis to strengthen our statements in this quantitative review (Fig. 2) [6].

**Table 1:** PICOTS system used for defining the review question and selection of eligible studies.

Population	D-R pairs in the context of kidney transplantation
Index prognostic factor	D-R SNP mismatch. Both, genome-wide or single locus mismatch were included
Comparator prognostic factors	Prognostic factors of interest were: HLA matching; HLA-eplet matching; sex; immunosuppression therapy
Outcomes	Graft loss or graft rejection were the only outcomes included in this review. Alternative outcomes such as eGFR slope, infections and death were not included
Timing	The retrospective evaluation of SNP mismatch between D and R did not have to occur at a defined time. No studies were excluded on the basis of duration of follow-up
Setting	D-R SNP mismatch was studied in a transplant setting. This information may be useful for a more personalized approach in immunosuppression for individual kidney transplant recipients in the future

eGFR, estimated glomerular filtration rate.

## RESULTS

### Overall D-R SNP mismatch

In an evolutionary context, SNPs can be regarded as being an omnipresent feature of all humans. Although genomic DNA sequence variations are created continuously at a rate of some 100 new single base changes per individual out of roughly 5 million SNPs per genome, most present-day human SNPs originated long after speciation, but before the emergence of different populations [8]. Thus, few of the SNP alleles that were present when humans emerged from Africa will have yet become fixed (reach 0% or 100%), with the consequence that human SNPs are generally not shared with our primate ancestors, but most are common to all human populations with only about 15% being “population-private” [8]. However, the small SNP-level mismatch between populations or even between subgroups (e.g. relatives) within a larger population may contribute a significant impact on organ D-R mismatching and alloimmunity.

Recent studies suggest that a lower SNP-level mismatch could be beneficial for graft outcomes in kidney transplantation. For instance, Reindl-Schwaighofer *et al*. genotyped 477 D-R pairs in a European transplant cohort [9]. D-R pairs were analyzed for their respective SNP mismatches in transmembrane or secreted proteins and stratified by quartiles. The degree of SNP mismatch was independently associated with graft loss in a multivariable model adjusted for HLA-eplet mismatch, with a hazard ratio (HR) of 1.68 [95% confidence interval (CI) 1.17–2.41] for every one unit of IQR increase. Furthermore, they found that as in HLA alloimmunity, donor-specific antibodies could be identified against genotype-derived non-HLA epitopes. Markkinen *et al*. validated the findings from Vienna in a kidney transplant cohort consisting of 1025 D-R pairs who underwent transplantation at the Helsinki University Hospital [9, 10]. In both studies, [a] SNP mismatch was defined as the donor carrying an allele that was not present in the recipient. Due to a small number of graft losses (*n* = 68) within the observational period (median follow-up 37 months), an alternative study outcome criterium with graft rejection was implemented (*N* = 199). It was reported that increasing mismatch sum in kidney-related proteins increased the risk for acute rejection with an unadjusted HR of 1.15 (95% CI 1.01–1.3) and an adjusted HR of 1.13 (95% CI 0.99–1.28) when dividing the mismatch sum into quartiles. Although the outcome criterium was different, this validates the results from Reindl-Schwaighofer *et al*. at least to an extent and underlines the connection between D-R SNP mismatch and transplantation outcomes [9]. Zhang *et al*. found that the proportion of genome-shared identity by descent (pIBD) was significantly associated with allograft survival, independent of HLA mismatches with a HR of 0.07 (95% CI 0.01–0.66) [11, 12]. The pIBD describes the proportion of shared inheritance of an identical portion of the whole genome between two individuals. Interestingly, they found an even stronger effect among 224 D-R pairs who were of Caucasian descent (HR 0.03; 95% CI 0.001–0.79), indicating the significance of the individual ancestry of donors and recipients even within the same race. The population for this study was drawn from the GoCAR (Genomics of Chronic Allograft Rejection) cohort, which included living and deceased donors [13]. Thus, the assumption could be made that the pIBD score is biased for living and deceased donors, since the probability of a living donor compared with a deceased donor being related with the recipient to a certain degree is much higher. Zhang *et al*. included a multivariable Cox regression analysis adjusted for donor type, which revealed that when dichotomizing the pIBD into pIBD < 0.1 and pIBD > 0.1, a pIBD < 0.1 was not significantly associated with graft loss in all D-R pairs (HR 2.04; 95% CI 0.66–6.33). The stratified analysis among all living donor recipients (*N* = 194) illustrated a similar trend with an HR of 1.67 (95% CI 0.47–5.92) for pIBD < 0.1. Nevertheless, in Caucasian-to-Caucasian D-R pairs (*N* = 224), the investigators found that D-R pairs with pIBD < 0.1 experienced more graft loss events than D-R pairs with pIBD > 0.1; however, the rather broad CI calls into question the accuracy of this assumption (HR: 5; 95% CI 0.93–26.7). Finally, we would like to note that in the above presented calculation of pIBD < 0.1 < pIBD, the exact value of 0.1 was not accounted for. In order to validate the main assertation of the study, a validation study should be conducted with a cohort consisting either exclusively of living or deceased donors. Sun *et al*. undertook a similar approach to Reindl-Schwaighofer *et* *al*., by defining mismatch as a donor carrying an allele that is not present in the recipient [9, 14]. To define gene-level mismatches, the mismatch status of all the variants within each annotated gene region were summed up and dichotomized. Investigation took place within two cohorts: GoCAR and CTOT (Clinical Trials in Organ Transplantation) [13, 15]. Interestingly, the authors found that the calculated gene level mismatch score highly correlated with the pIBD (*P* < .001) used by Zhang *et al*., which adds comparability to the publication mentioned earlier in this chapter [11]. In addition, Sun *et al*. showed that the normalized mismatch score could reflect the relative differences in genetic ancestries of D-R pairs in both cohorts, where inter-ancestry pairs generally had larger mismatch scores than intra-ancestry pairs, highlighting the impact of ancestry on genome-wide mismatch [14]. Furthermore, the investigators found that after adjusting for HLA mismatch score, induction therapy and donor status, increased genome-wide mismatch scores were associated with death-censored graft loss (DCGL) in the larger discovery cohort (GoCAR), with [a] HR of 1.25 (95% CI 1.01–1.56), which was validated in the smaller CTOT cohort, although without numerical significance due to a smaller sample size and fewer outcomes [14]. Cao *et al*. used a different approach to examine the association of SNP mismatch and graft rejection in kidney transplantation [16]. In contrast to Reindl-Schwaighofer *et al*., Markkinen *et al*. and Sun *et al*., Cao *et al*. defined SNP mismatch as any mismatch between donor and recipient, regardless of directionality [9, 10, 14, 16]. This identity by state (IBS) score was then utilized to calculate a polygenic risk score (PRS) in one cohort [DeKAF (Deterioration of Kidney Allograft Function)] and subsequently validated in another cohort [GEN-03 (Genomics of Kidney Transplantation); dbGaP Study Accession: phs001667.v1.p1] [17]. It should be noted that both cohorts consisted exclusively of living donors. The results showed that the estimated HR for the PRS was 1.54 (95% CI 1.07–2.22), thus indicating significant correlation between the IBS-PRS score and acute graft rejection. Finally, it should be noted that Reindl-Schwaighofer *et al*. examined exonic, non-synonymous SNPs coding for transmembrane or secreted proteins which Markkinen *et al*. validated, whereas Zhang *et al*., Sun *et al*. and Cao *et al*. studied the association of genome-wide SNP mismatch, both exonic and intronic, with graft rejection respectively graft loss [9, 10, 11, 14, 16].

The presented results of the publications discussed in this chapter indicate a strong correlation between genome-level mismatch and either graft loss or graft rejection events. Figure 2 displays the forest plot and the meta-analysis of the identified studies. Although overall non-HLA SNP mismatch clearly contributes to kidney transplantation attrition, the question remains as to whether kidney transplant patients can benefit from this newly gained knowledge. As whole-genome sequencing becomes cheaper every year, the possibility of matching D-R pairs according not only to HLA but also to non-HLA genetic traits may potentially facilitate new approaches in kidney transplant precision medicine in the near future [18]. Specifically, adjustment of maintenance immunosuppression based on HLA and non-HLA mismatch scores might be the most promising and feasible advance [19].

### Locus-specific mismatches

#### LIMS1 (a bridge linking integrin-linked kinase to NCK adaptor protein 2 which links nephrin to actin in kidney podocytes)

As indicated in the previous paragraph, genome wide SNP-mismatch scores proved to be significant predictors for graft outcomes. However, the mismatch calculation must be conducted in every plausible D-R pair during the kidney allocation process, thus resulting in high cost and low efficiency per patient. A locus-specific approach, where patients could be matched depending on a single SNP, would be much more efficient. Steers *et al*. conducted a two-stage study in 2019 [20]. In the discovery phase, 50 deletion tagging SNPs were examined in a Kaplan–Meier analysis in 705 recipients who underwent transplantation at Columbia University Irving Medical Center in New York. One single SNP (rs893403) at the *LIMS1* gene locus surpassed Bonferroni-corrected significance. In the replication phase, rs893403 was genotyped in kidney recipients and their respective donors in three transplant cohorts (Belfast, Torino and TransplantLines), resulting in an overall number of 2004 genotyped D-R pairs. In the combined D-R analysis, a genomic collision (mismatch) was defined as a specific D-R genotype combination in which a recipient who was homozygous for a deletion-tagging allele received a transplant from a non-homozygous donor. The intronic copy number variation (CNV)-tagging SNP (rs893403) resulted in lower *LIMS1* mRNA expression levels in homozygous carriers [20]. This is similar to a loss-of-function variant, where the epitope is not expressed in the recipient but in the allograft, thus facilitating an immune response against the newly introduced epitope [21]. In the combined stratified analysis of the 2004 D-R pairs across three cohorts, the recipients originating from D-R pairs with the collision genotype had a 58% greater risk of allograft rejection than D-R pairs without the collision genotype, with a significant HR of 1.58 (95% CI 1.27–1.97). Furthermore, the sole homozygosity for the risk allele (rs893403) in the recipients, regardless of the donor *LIMS1* allele status, was also deemed as a significant predictor of higher risk of graft rejection (HR 1.41; 95% CI 1.14–1.74). On top of that, the investigators demonstrated in a subgroup analysis that *LIMS1* alloresponse was most specific to homozygous-risk patients with graft rejection (*P* = .002). Caliskan *et al*. attempted to replicate the results of Steers *et al*. in 2021 [20, 22]. They genotyped 841 kidney transplant recipients being followed at the Istanbul School of Medicine Transplant Clinic, in order to examine the correlation between recipient homozygosity for the risk allele and long-term allograft survival. In addition, 41 recipients were genotyped together with their donors to facilitate a more exact definition of the collision genotype theory in a subgroup analysis. No significant correlation between the rs893403 GG risk genotype and graft loss (HR 0.97; 95% CI 0.68–1.38) could be found. Despite this negative result, the group reported a higher incidence of T-cell-mediated rejection (TCMR) in the GG-group, when compared with the AG/AA group [25 (12.5%); 35 (5.5%); *P* = .001] after a median follow-up of 11.4 years. There was no difference in antibody-mediated rejection (ABMR) between the two groups. Interestingly, in 2022 Markkinen *et al*. also wanted to replicate the findings of Steers *et al*., but could not find any association between the *LIMS1* SNP rs893403 and graft rejection [10, 20]. Neither the D-R collision analysis (HR 0.85; 95% CI 0.48–1.50), nor the recipient-only (rs893403) analysis (HR 0.78; 95% CI 0.45–1.38) revealed a significant correlation with graft failure. However, the authors found a significant correlation between another SNP polymorphism and graft rejection: It was observed that a deletion-tagging mismatch at the rs7542235 locus [which has been reported to tag for deletions in Complement factor H–related proteins (*CFHR*) proteins 1–3] was significantly associated with a higher risk for rejection when compared with the no mismatch status with an adjusted HR of 2.97 [10]. Factor H–related proteins are plasma proteins that bind to the complement component C3b. Mutations in individual *CFHR* genes are associated with diseases such as C3 glomerulopathies [23]. However, we view these results rather critically, as only 16 patients out of the 1025 study participants were homozygous-risk allele carriers for rs7542235, resulting in a wide CI (95% CI 1.46–6.05). It should be further noted that the median follow-up in the Markkinen study was 37 months, which is shorter than the 8.6 years of follow-up analyzed in Steers *et al*. and 11.4 years in Caliskan *et al*. [20, 22]. Sun *et al*. conducted a study in 2023 in which they scanned the whole genome in an effort to pinpoint specific non-HLA gene loci at which D-R mismatches are associated with graft loss [14]. To achieve this, they derived gene-level mismatch scores by summing over variant-level mismatch scores for variants mapped to each gene region. They found that on a gene level, D-R mismatches at the *LIMS1* gene locus were associated with DCGL (HR 2.21; 95% CI 1.32–3.70) in the GoCAR discovery cohort, when adjusted by genome-wide mismatch, donor-specific antibody, induction therapy, donor status and HLA mismatch [13]. The CTOT [15] (*N* = 146) validation cohort indicated a similar trend with a HR of 5.35. However, the 95% CI for the presented HR ranged from 1.26 to 22.71, which indicates a rather low degree of precision in the estimation of the parameter. Sun *et al*. examined the association between DCGL and *LIMS1* not only on a genome level, but furthermore on a variant level [14]. The variant-level analysis showed an association between DCGL and the *LIMS1* risk variant rs893403 (presence of homozygosity in the CNV-tagging minor G-allele in the recipient and the presence of the major allele in the donor kidney). D-R pairs with the collision genotype showed an increased risk of DCGL in the GoCAR cohort (*N* = 384) with an HR of 2.33 (95% CI 1.19–4.57), adjusted by genome-wide mismatch, HLA mismatch score, donor status and induction therapy. Finally, Caliskan *et al*. published another paper in 2024 examining the *LIMS1* locus in the context of kidney transplantation [24]. A total of 110 transplant recipients who were followed at the Istanbul Faculty of Medicine Transplant Clinic were included in the study. The study population in this study differed from the other cohorts discussed in this paper since only patients who underwent for-cause kidney biopsy due to an increase in creatinine serum levels or proteinuria were included in the study. Recipients were categorized based on their *LIMS1* rs893403 variant genotype GG vs AG/AA. There was no significant difference regarding acute rejection (ABMR or TCMR) between the two groups, with an odds ratio (OR) of 1.58 (95% CI 0.61–4.09). Although it was shown that the mean Banff tubulitis score correlated significantly with the risk genotype (*P* = .03), several other endpoints that were also examined did not show significance. Since multiple testing occurred without application of Bonferroni correction or other stratification for multiple testing, we regard these results rather critically.

### *MICA* and IL6 mismatch

Besides *LIMS1*, there is an abundance of other genetic loci which are subject to studies in the context of kidney transplantation. However, most publications only explore direct correlations between recipient SNPs and various outcomes such as graft loss, rejection or Banff scores, without shedding light on the D-R SNP mismatch aspect, which we are principally interested in [25–27]. In fact, we identified three additional studies during the extensive literature search, fitting the subject of SNP D-R mismatch, none of which investigated the *LIMS1* locus apart from Markkinen *et al*., who found an association between the deletion-tagging mismatch in the rs7542235 genotype, which was already discussed in the previous section [10]. Eskandari *et al*. examined the correlation between interleukin 6 (IL6)/IL6 receptor (IL6R)/IL10/IL10RA-B polymorphisms and 15-year death-censored allograft survival [28]. A total of 1271 patients who received a single-kidney transplant at the University Medical Center Groningen and their respective donors were included and genotyped for the respective eight candidate-SNPs of interest, i.e. *IL6* (*rs1800795*), *IL6R* (*rs2228145*), *IL10* (*rs1800871, rs1800896* and *rs3024498*), *IL10RA* (*rs2229113* and *rs3135932*) and *IL10RB* (*rs2834167*). Notably, they found that the IL6 SNP rs1800795 was associated with lower death-censored allograft loss. The mismatch in this study differs from the collision genotype model which we explained in connection with *LIMS1*. D-R pairs were divided into three groups: (i) neither the donor nor the recipient having the C|C-genotype, (ii) either the donor or the recipient having the C|C genotype, or (iii) both the donor and the recipient having the C|C genotype. In a multivariable analysis, the combined C|C-genotype in D-R pairs was significantly associated with death-censored graft loss with a HR of 0.65 (95% CI 0.48–0.88), after adjusting for confounders. This finding fits the propositions of studies that have been discussed in the “Overall D-R mismatch” section of this review, which indicate that lower overall D-R mismatch is associated with better allograft survival/lower allograft loss. The question remains whether the IL6 SNP mismatch investigated by Eskandari *et al*. has a disproportionately greater impact on graft survival than other SNPs, which needs to be validated in further studies [28]. IL10 SNPs were not tested for actual D-R mismatch but rather examined for their individual impact on graft survival in the donors and recipients individually. Notably, no significant difference in 15-year death-censored graft loss for any of the IL10 and IL10R SNPs in the transplant donors or recipient was found.

Carapito *et al*. investigated the relationship between *MHC class I polypeptide-related sequence A* (*MICA*) allele D-R mismatches and graft survival [29]. *MICA* encodes the highly polymorphic major histocompatibility complex class I chain–related protein A. The protein product is expressed on the cell surface and functions as a stress-induced antigen that is broadly recognized by intestinal epithelial gamma-delta T cells [31]. The analysis involved 1356 patients and their respective donors from six French transplant centers (Montpellier, Paris-Saint-Louis, Toulouse, Paris-Necker, Nancy and Nantes). They found that after a median follow-up of 6.3 years, *MICA* allele D-R mismatch (single or double mismatch) was an independent factor associated with graft loss (HR 2.12; 95% CI 1.45–3.11). Littera *et al*. conducted a similar analysis in a cohort consisting of 148 Sardinian patients who underwent kidney transplantation at the Organ Transplantation Center of the G. Brotzu Hospital in Cagliari, Italy [31]. The cohort was subdivided into 68 patients who showed ABMR in an allograft biopsy and 80 randomly selected transplant recipients with stable graft function. The median follow-up time period was 52.9 months for *MICA*-matched patients and 64.7 months for *MICA*-mismatched patients. At 5 years post-transplantation, graft loss was 20.8% for *MICA*-matched patients and 35.5% for *MICA*-mismatched patients (OR 2.09; 95% CI 0.73–5.98). The broad confidence interval is a result of the rather small sample size. The authors further reported that compared with *MICA*-mismatched patients, those matched for *MICA* alleles exhibited a significantly reduced risk of ABMR after 5 years (Log-rank *P* = .03). In conclusion, assessment of *MICA* matching and immunization for the identification of patients at high risk for transplant rejection and loss is warranted, but it is of note that *MICA* is highly associated with HLA class I genes (*MICA* gene is located, within the HLA complex, 46 kb centromeric to the HLA-B locus, *MICA*-HLA-B haplotype diversity and linkage disequilibrium), and thus an underlying HLA-effect cannot be ruled out.

## CONCLUSIONS

Spurred on by the goal of reaching better long-term allograft outcomes for kidney transplant recipients, some researchers have expanded their view from a solely HLA-centered matching approach and are starting to take a broader spectrum of pathophysiologic mechanisms behind kidney allograft failure/rejection into account. In 2016, Mesnard *et al*. lay the foundation in their study of non-HLA D-R mismatch, when they showed that the calculated allogenomics mismatch score between donors and recipients were significantly associated with eGFR [32]. Despite the small sample size and therefore the alternative outcome parameter (estimated glomerular filtration rate), this study was the first to investigate this topic of non-HLA alloimunity and got the ball rolling for all further studies that were published from 2019 onwards [9, 20]. In this quantitative review, we discuss recent publications describing the impact of non-HLA SNP mismatch on graft outcomes. The current literature clearly shows that overall SNP mismatch represents an independent predictor for graft outcomes. These findings strengthen the hypothesis that there are relevant immunological mechanisms outside of just HLA mismatch in kidney transplantation outcomes. Although some papers did not find an association, the overall data suggests that the collision genotype between D-R pairs at the *LIMS1* locus also correlates with worse graft outcomes (Fig. 2) [10, 22, 25]. It is worth emphasizing that the *LIMS1* SNP of interest in the reviewed papers is intronic. Furthermore, while the directionality of rs893403 is the key factor identified in multiple studies and associated with a collision pattern, the culprit gene—whether *LIMS1* or adjacent *GCC2*—as well as culprit mechanism remain not fully understood. A thorough elucidation of this mechanism is essential for the effective development of precision medicine strategies targeting this locus. Ongoing research on this topic, especially the identification of further single non-HLA loci that disproportionately influence graft outcomes, may facilitate the construction of a risk score that consists of single loci, which can predict better or worse graft outcomes in individual D-R settings. This would allow for a precision medicine approach in the guidance of immunosuppression. Currently, there are efforts to improve personalized immunosuppression in accordance with HLA-eplet mismatch. The ongoing research progress in the non-HLA domain might facilitate a combined non-HLA and HLA approach regarding personalized immunosuppression in the near future. We therefore encourage further research on the topic of non-HLA mismatch in kidney transplantation.

## Data Availability

Most of the data used to write this quantitative review are publicly available and referenced in the manuscript. In one exceptional case, we did get in contact with the corresponding author of an original article to obtain additional information about the paper.
